# A new class of broadly neutralizing antibodies that target the glycan loop of Zika virus envelope protein

**DOI:** 10.1038/s41421-019-0140-8

**Published:** 2020-02-04

**Authors:** Panke Qu, Chao Zhang, Min Li, Weimin Ma, Pei Xiong, Qingwei Liu, Gang Zou, Dimitri Lavillette, Feifei Yin, Xia Jin, Zhong Huang

**Affiliations:** 1 CAS Key Laboratory of Molecular Virology & Immunology, Institut Pasteur of Shanghai, Center for Biosafety Mega-Science, Chinese Academy of Sciences, University of Chinese Academy of Sciences, Shanghai, 200031 China; 2 Hainan Medical University-The University of Hong Kong Joint Laboratory of Tropical Infectious Diseases, Hainan Medical University, Haikou, Hainan, 571101 China; 3 Key Laboratory of Translation Medicine Tropical Diseases, Department of Ministry of Education, Hainan Medical University, Haikou, Hainan, 571101 China

**Keywords:** Immunology, Biological techniques

## Abstract

Zika virus (ZIKV) infection poses a serious threat to human health. However, no licensed vaccine or therapeutic drug is currently available for ZIKV. We have previously shown that recombinant ZIKV E80 protein induced potent neutralizing antibody response and protected mice from lethal viral challenge. In the present study, we isolated five ZIKV neutralizing monoclonal antibodies (mAbs) from E80-immunized mice. These five mAbs specifically bound and neutralized Asian-lineage ZIKV strains. Epitope mapping revealed that all of the five mAbs recognized a novel linear epitope located on the glycan loop of E protein domain I. Sequence alignment revealed that the epitope was extremely conserved in ZIKV but highly variable between ZIKV and other flaviviruses. Thus, these five mAbs form a new class of anti-ZIKV antibodies exhibiting broad-spectrum neutralization on Asian-lineage ZIKV. A representative of this mAb class, 5F8, was found to exert inhibitory function in vitro primarily at the early stage of the post-attachment viral entry process. Importantly, mAb 5F8 was able to confer full protection in a mouse model of ZIKV lethal infection. Our results have strong implications for developing anti-ZIKV vaccines and therapeutic mAbs.

## Introduction

Zika virus (ZIKV) is a member of the Flavivirus family and is transmitted by mosquitoes. It was first discovered in monkey in Uganda’s Zika Forest in 1947^1^ and first reported to cause human diseases in 1952^2^. After being quiescent for 60 years, large outbreaks of ZIKV infection have been reported in some regions/countries such as Yap^3,4^, French Polynesia^5,6^, Brazil^7^, and quickly spread globally in 2016. Besides mild symptoms and signs including fever, rash, joint pain, or conjunctivitis^8,9^, severe brain abnormalities such as microcephaly were linked to ZIKV infection in recent outbreaks^10–12^. In experimental models, ZIKV infection is indeed able to cause microcephaly and other birth defects in mice^13–15^, and in non-human primates^16^. Despite ZIKV poses a serious threat to public health, there are currently no licensed vaccines or therapeutic drugs to prevent or treat ZIKV infection.

Like other flaviviruses, ZIKV is an enveloped virus and possesses a ~11 kb single-stranded positive-sense RNA genome. This viral genome encodes a large polyprotein precursor that is subsequently processed by host and viral proteases into three structural proteins (capsid [C], premembrane/membrane [prM/M], and envelope [E] proteins) and seven nonstructural proteins^17^. Mature ZIKV virion is ~50 nm in diameter and has a smooth outer shell made of 90 dimers of E and M proteins^18,19^. ZIKV E protein consists of an ectodomain and a stem/transmembrane domain, and it has a single N-linked glycosylation site on residue N154 which is located on the glycan loop (also known as “150 loop”; containing residues 145 to 165)^18–20^. The ectodomain can be further divided into three distinct subdomains, namely EDI, EDII, and EDIII^17–19^.

Neutralizing antibodies play a critical role in protection against ZIKV infections^21–24^. Therefore, gaining comprehensive knowledge on the landscape of neutralizing antibody epitopes is important for design and development of anti-ZIKV vaccines^25–27^. Recently, human or murine monoclonal antibodies (mAbs) exhibiting neutralization effect on ZIKV have been identified and characterized by a number of groups^20,23,24,28–33^. Similar to other flaviviruses, E protein of ZIKV is the main antigen targeted by neutralizing antibodies^25–27^. Binding epitopes for anti-ZIKV neutralizing mAbs have been located to either single EDIII or single EDII subdomains^20,23,29–31^. In addition, tertiary/quaternary epitopes, which involve two distinct subdomains within the same protomer or two identical subdomains from different protomers have been identified^29,32,33^. Neutralizing mAbs that bind EDIII are usually ZIKV-specific^23,30,31^, whereas anti-ZIKV mAbs targeting the fusion loop on EDII are cross-reactive with closely related flaviviruses such as dengue virus (DENV) and may therefore lead to antibody-dependent enhancement (ADE) of DENV infections^20,23,29–31^. Thus far, neutralizing mAbs that solely bind ZIKV EDI subdomain have not been reported.

Recently, our group produced the ectodomain (termed E80) of ZIKV E protein in insect cells and further demonstrated that the recombinant E80 protein potently elicited neutralizing antibodies in mice^34^. In the current study, we generated five neutralizing mAbs from E80-immunized mice and subsequently characterized them by using a variety of in vitro and in vivo assays. Interestingly, all of the five mAbs were found to neutralize only the Asian-lineage ZIKV strains and bind to the same linear epitope located on the glycan loop of EDI, thus forming a unique group of anti-ZIKV neutralizing mAbs. One representative mAb in this group, 5F8, was used to demonstrate protection in mice against lethal ZIKV challenge. These findings enhance our understanding of ZIKV-specific neutralizing antibody epitopes and protective immunity, thus having strong implication for development of recombinant ZIKV vaccines and mAb therapeutics.

## Results

### Generation of ZIKV neutralizing mAbs

Splenocytes obtained from ZIKV E80-immunized mice were fused with SP2/0 myeloma cells to generate hybridomas. Culture supernatants from the resulting hybridomas were first screened by ELISA for their reactivity to ZIKV E80 protein. Thirty individual hybridoma clones were found to be ELISA-positive (Supplementary Fig. S1). Then, supernatants of these 30 clones were evaluated for their ability to neutralize ZIKV strain SZ-WIV01 by standard neutralization assay. Five clones (3E8, 5F8, 5G3, 8A2, and 9C3) were found to strongly neutralize ZIKV/SZ-WIV01 (Table 1), whereas the other 25 clones (such as 1C11 and 4C5 listed in Table 1) did not show any neutralizing activity. Our subsequent analyses focused on the five neutralizing mAbs.

**Table 1:** Characteristics of the anti-ZIKV mAbs.

		Supernatant		Purified mAbs					
MAb^a^	Isotype^b^	ELISA^c^	Neutralization^d^	Affinity for E80 K_D_ (nM)^e^	PRNT50 (µg/ml)				
					ZIKV/SZ-WIV01	ZIKV/PRVABC-59	ZIKV/FLR	ZIKV/MR766	DENV2/NGC
1C11	IgG2a	+	–	NA^f^	>800	NA	NA	NA	NA
4C5	IgG1	+	–	NA	>800	NA	NA	NA	NA
3E8	IgG1	+	+	5.4 ± 1.3	6.7	9.7	63.8	>800	>800
5F8	IgG1	+	+	15 ± 3.1	5.7	27.1	41.6	>800	>800
5G3	IgG1	+	+	8.3 ± 2.2	7.1	27.6	109.6	>800	>800
8A2	IgG1	+	+	6.9 ± 2.3	10.5	42.1	176.7	>800	>800
9C3	IgG1	+	+	2.5 ± 0.5	26.0	128.2	438.8	>800	>800
EV71 D5	IgG1	–	–	NA	>800	>800	>800	>800	>800

^a^30 hybridoma clones were obtained by immunizing mice with ZIKV E80 protein, and 7 out of them (1C11, 4C5, 3E8, 5F8, 5G3, 8A2, and 9C3) were shown in the table

^b^MAb isotype was determined by SBA Clonotyping^TM^ System/HRP ELISA kit

^c^Culture supernatants from hybridomas were screened by ELISA for their reactivity to ZIKV E80 protein, and results were expressed as follows: +, OD450 > 0.9; –, OD450 < 0.15

^d^Culture supernatants from hybridomas were evaluated for their capacity to neutralize ZIKV/SZ-WIV01 using neutralization assay, and results were expressed as follows: +, >50% of plaque formation was inhibited; –, no significant inhibition of plaque formation

^e^*K*_D_ (equilibrium) for the ZIKV E80/mAb interaction was determined by BLI

^f^NA, not assessed

### Binding characteristics of the neutralizing mAbs

**Fig. 1:**
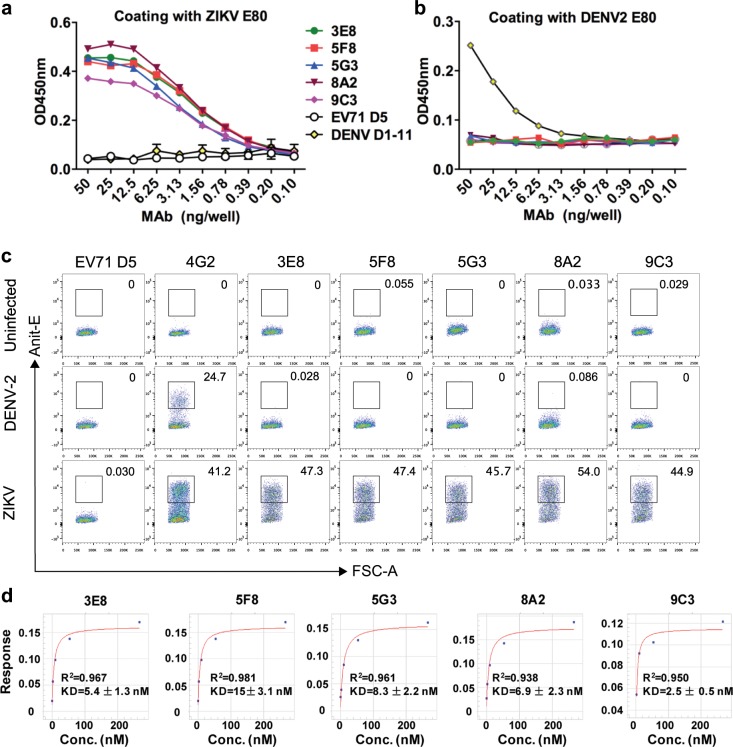
Binding specificity and affinity of the mAbs to ZIKV E protein. **a**, **b** Reactivities of anti-ZIKV mAbs (3E8, 5F8, 5G3, 8A2, 9C3) to the E protein ectodomains (E80) of ZIKV (**a**) and of DENV2 (**b**) measured by ELISA. Anti-EV71 mAb D5 was used as a negative control. Anti-DENV mAb D1-11 served as a positive control for detection of DENV2 E80 protein. Error bars represent standard error of the mean (SEM). **c** Flow cytometry analysis of reactivities of the mAbs with ZIKV and DENV viruses. ZIKV/SZ-WIV01- and DENV2/NGC-infected Vero cells were incubated with anti-ZIKV mAbs, negative control mAb D5 or flavivirus cross-reactive mAb 4G2 (positive control), and then analyzed by flow cytometry. **d** Binding affinities of the mAbs towards ZIKV E80 determined by biolayer interferometry (BLI). KD, equilibrium dissociation constant between the antibody and its antigen. Conc. is the abbreviation for concentration.

To evaluate the binding specificity of the five neutralizing mAbs, we performed ELISA with either recombinant ZIKV E80 or recombinant DENV2 E80 protein as coating antigen. All of the five anti-ZIKV mAbs (3E8, 5F8, 5G3, 8A2, and 9C3) were found to react with ZIKV E80 protein in an antibody dose-dependent manner (Fig. 1a), whereas none of them showed binding activity to DENV2 E80 protein regardless those doses of mAb used (Fig. 1b), indicating that the five mAbs specifically bind ZIKV E80. Next, we performed flow cytometry analysis to determine whether the five mAbs also specifically recognize ZIKV in the context of viral infection. As controls, an irrelevant mAb D5 (against enterovirus 71 (EV71)^35^ produced no or only baseline level of signal in ZIKV- or DENV-infected cells whereas a known flavivirus cross-reactive mAb 4G2^36^ recognized both DENV2- and ZIKV-infected Vero cells (Fig. 1c), thus validating the assay. As shown in Fig. 1c, each of the five mAbs (3E8, 5F8, 5G3, 8A2, and 9C3) could positively stain ZIKV-infected cells but not DENV2-infected cells. These data demonstrate that the five anti-ZIKV E80 mAbs were indeed specific for ZIKV and had no cross-reactivity with DENV2.

Biolayer interferometry (BLI) assays were performed to determine the binding affinity of the five neutralizing mAbs towards recombinant ZIKV E80 protein. As shown in Fig. 1d, 3E8, 5F8, 5G3, 8A2, and 9C3 mAbs exhibited high binding affinities to ZIKV E80 with equilibrium dissociation constants (KD) of 5.4, 15, 8.3, 6.9, and 2.5 nM, respectively.

### The five mAbs specifically recognized and neutralized Asian-lineage ZIKV strains

**Fig. 2:**
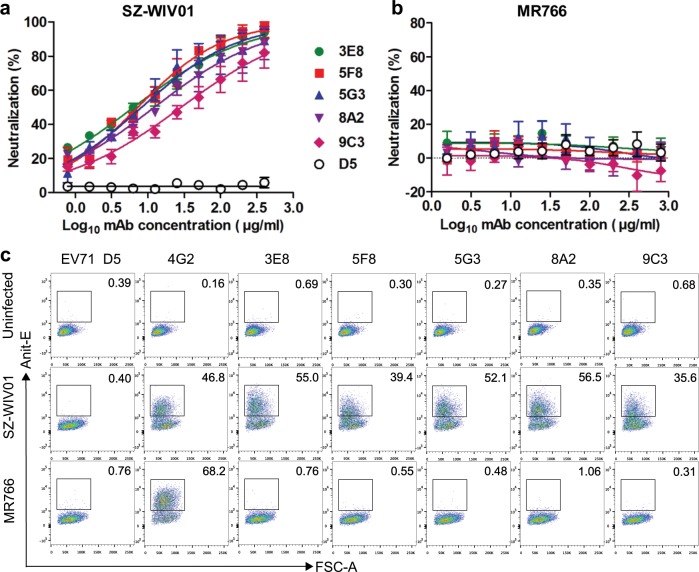
The mAbs specifically neutralized and recognized Asian-lineage ZIKV strain. **a**, **b** Neutralization activity of the mAbs against Asian-lineage (**a**) and African-lineage (**b**) strains of ZIKV. 100 PFU of Asian ZIKV strain SZ-WIV01 (**a**) or African ZIKV strain MR766 (**b**) was mixed with two-fold serial dilutions of purified anti-ZIKV mAbs 3E8, 5F8, 5G3, 8A2, 9C3 or control mAb D5. Then neutralizing activity of these mAbs was determined by the plaque reduction neutralization test (PRNT). Results represent the mean ± SEM of three independent experiments. **c** Flow cytometry analysis of reactivities of the mAbs with Asian and African strains of ZIKV. ZIKV/SZ-WIV01- and ZIKV/MR766-infected Vero cells were incubated with anti-ZIKV mAbs, negative control mAb D5 or positive control mAb 4G2, and then analyzed by flow cytometry.

We performed neutralization assays to determine the neutralization capacity of the five mAbs against a panel of representative viruses, including the homologous strain ZIKV/SZ-WIV01 (Asian lineage), strain ZIKV/PRVABC-59 (Asian lineage), strain ZIKV/COL/FLR/2015 (FLR, Asian lineage), strain ZIKV/MR766 (African lineage), and DENV2 strain New Guinea C (NGC)^37^. The results showed that 3E8, 5F8, 5G3, 8A2, and 9C3 mAbs potently neutralized the homologous strain ZIKV/SZ-WIV01 with 50% plaque reduction neutralization titers (PRNT50) of 6.32, 7.21, 8.17, 12.19, and 28.44 µg/ml, respectively (Fig. 2a). These five mAbs also exhibited varying degrees of cross-neutralizing activity against the other two Asian-lineage ZIKV strains, PRVABC-59 and COL/FLR/2015 (FLR), but failed to neutralize African-lineage prototype strain MR766, nor did they neutralize the DENV2/NGC strain (Table 1 and Fig. 2b). These data indicate that the mAbs 3E8, 5F8, 5G3, 8A2, and 9C3 specifically neutralized Asian-lineage ZIKV strains.

To investigate why the five mAbs failed to neutralize the African-lineage ZIKV strain MR766, we stained MR766- and SZ-WIV01-infected cells with the mAbs and subsequently performed flow cytometry analysis. As expected, the flavivirus cross-reactive antibody 4G2 produced positive signals in both SZ-WIV01- and MR766-infected cells (Fig. 2c), validating the assay. Following treatment with each of the five mAbs (3E8, 5F8, 5G3, 8A2, and 9C3), positive signal was detected only in the SZ-WIV01-infected samples, but not in the MR766-infected ones. These results indicate that mAbs 3E8, 5F8, 5G3, 8A2, and 9C3 could not recognize and bind MR766, resulting in the lack of neutralization potency on MR766.

### Mapping of mAb epitopes

**Fig. 3:**
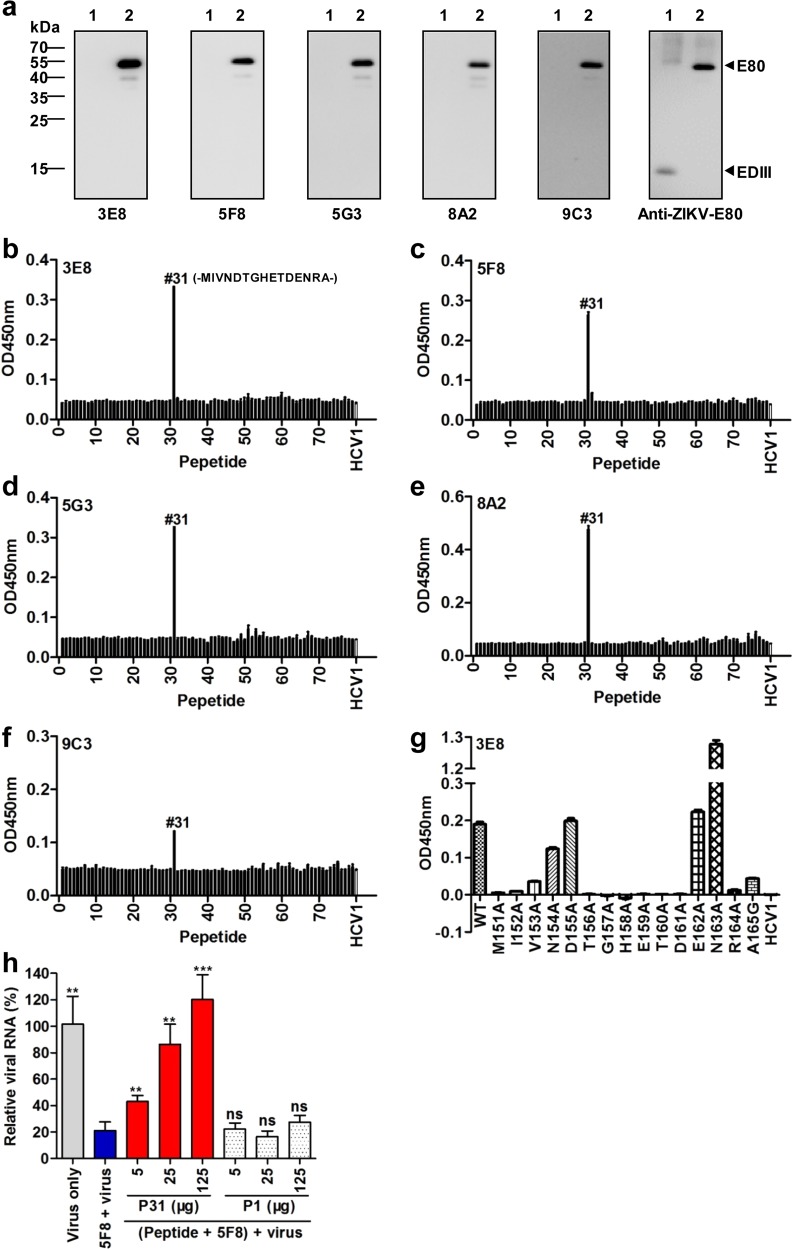
Epitope mapping of anti-ZIKV mAbs. **a** Reactivities of anti-ZIKV mAbs with ZIKV E80 and EDIII antigens determined by western blotting. Anti-ZIKV-E80 sera served as positive control. Lane 1, ZIKV EDIII; lane 2, ZIKV E80. **b**–**f** Reactivities of anti-ZIKV mAbs 3E8 (**b**), 5F8 (**c**), 5G3 (**d**), 8A2 (**e**) and 9C3 (**f**) with E80 peptides measured by peptide-ELISA. A set of 79 overlapping peptides spanning the whole sequence of E80 of ZIKV were coated to the plates. An irrelevant HCV peptide HCV1 served as a negative control. **g** Fine epitope mapping of mAb 3E8 by scanning mutagenesis. Fifteen peptides with a single amino acid substitution at each position of peptide #31 (designated P31) were tested for their reactivities with 3E8 by peptide ELISA. WT, wild type P31. Peptide variant nomenclature: first letter = original amino acid; number = position in ZIKV E protein; second letter = mutant amino acid. Error bars represent SEM. **h** Neutralization-inhibition assay. MAb 5F8 was preincubated with peptides P31 or P1 (control) for 1 h before neutralization assay. Intracellular viral RNA levels were detected by real-time PCR at 16 h post-infection. For each treatment, viral RNA level relative to that for the virus only group is shown. Data are mean ± SD of triplicate wells. Viral RNA levels of the peptide treatment groups were compared with that in the 5F8 + virus group, and statistical significance was indicated as follows: ns., no significant difference (*P* ≥ 0.05); **P < 0.01; ***P < 0.001.

We firstly performed Western blot assays to determine whether the five anti-ZIKV mAbs recognized recombinant ZIKV E80 and EDIII proteins on the blots. Anti-ZIKV-E80 mouse sera were used as positive control in the assays^34^. As shown in Fig. 3a, anti-ZIKV-E80 sera reacted with both EDIII and E80; whereas the 3E8, 5F8, 5G3, 8A2, and 9C3 mAbs were able to strongly detect E80, but failed to react with EDIII. The results indicate that these mAbs recognize linear epitopes which may reside in EDI and/or EDII.

Next, the mAbs were screened by ELISA for reactivity with a set of 79 overlapping synthetic peptides that spanned the entire ZIKV E80 region. As shown in Fig. 3b–f, all of the five mAbs strongly reacted with the peptide #31 (MIVNDTGHETDENRA, designated P31), which corresponds to residues 151 to 165 of ZIKV E protein (numbering according to the sequence of ZIKV strain Z1106033)^38^. These results indicate that the five mAbs share the same binding epitope within the P31 peptide (hereafter denoted as the “P31” epitope).

Then, we performed scanning mutagenesis to identify key residues within the epitope. Each amino acid in P31 was separately mutated to alanine (A) except that residue A165 was substituted to glycine (G). The original and mutated peptides were assessed for mAb binding by ELISA. In the case of mAb 3E8, replacement of N154, D155, and E162 by alanine did not influence the mAb-peptide binding, whereas V153A and A165G mutations exhibited significantly decreased 3E8 binding activity (Fig. 3g). Most notably, substitutions of M151, I152, T156, G157, H158, E159, T160, D161, and R164 with alanine completely or near-completely abolished the interaction of mAb 3E8 with P31 (Fig. 3g), indicating that these residues were critical for mAb binding. Interestingly, an alanine substitution at N163 significantly increased the binding of mAb 3E8 to P31 (Fig. 3g). For the 5F8, 5G3, 8A2, and 9C3 mAbs, their binding profiles to P31 mutants were similar to that of 3E8 mAb (Supplementary Fig. S2), confirming that these five mAbs bind the same “P31” epitope.

To verify the function of the “P31” epitope, we performed neutralization-inhibition assay. In this assay, peptides P31 and P1 (control) were separately mixed with a representative of the mAb class, 5F8, for 1 h prior to neutralization assay. For each treatment, the amounts of viral RNA in cells were determined by quantitative RT-PCR at 16 h post-infection. As shown in Fig. 3h, mAb 5F8 significantly inhibited viral infection. Pretreatment with peptide P1 (control) did not affect the neutralizing activity of mAb 5F8 regardless of the peptide dose. By contrast, preincubation with peptide P31 resulted in significant increase in viral RNA levels, suggesting that P31 has a dose-dependent neutralization-inhibitory activity. These results demonstrate that the identified epitope “P31” indeed serves as a neutralizing antibody epitope.

**Fig. 4:**
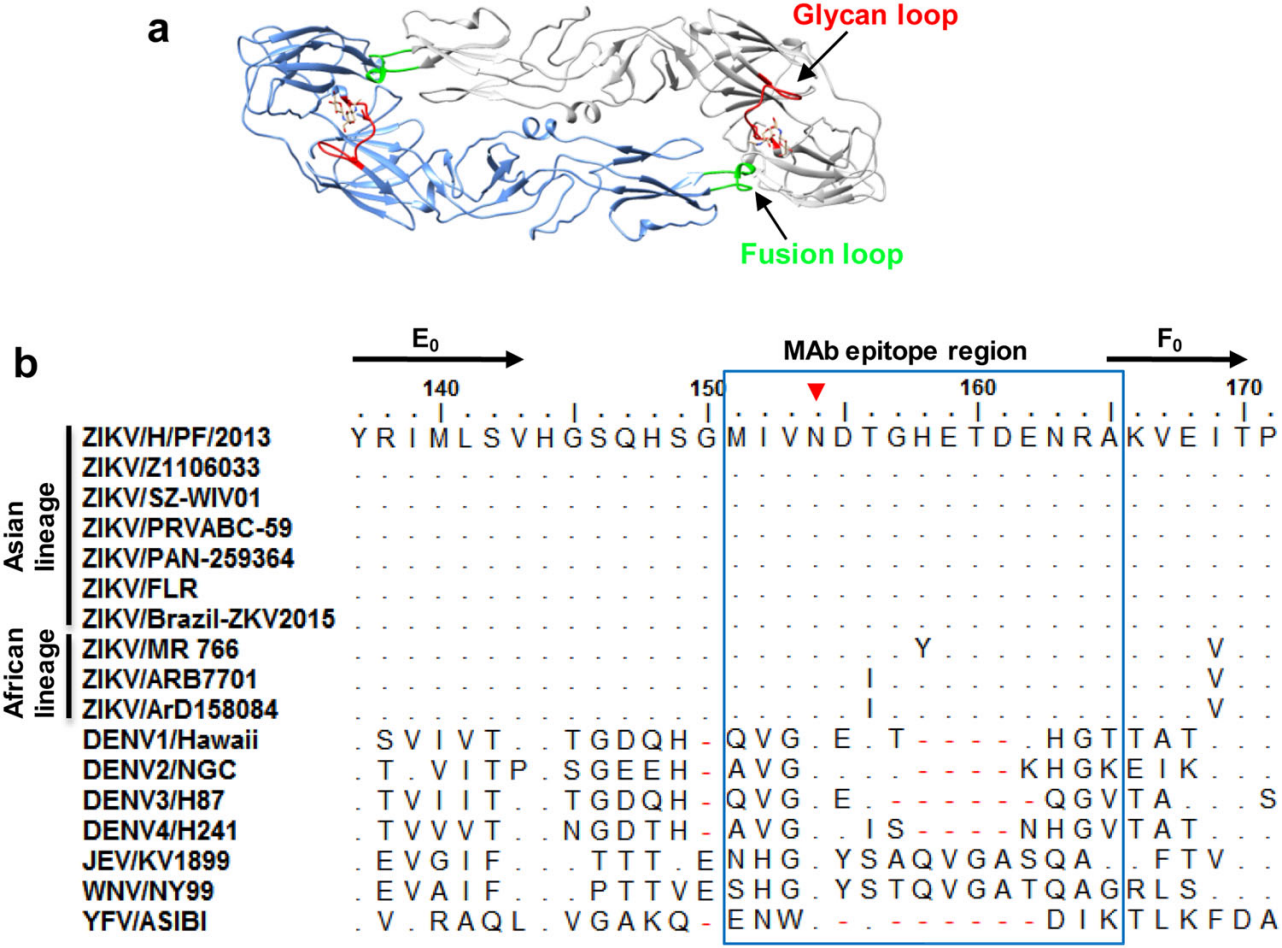
Location and sequence of the P31 epitope. **a** Ribbon diagram of top view of the E protein dimer. The fusion loop (green) from one monomer (cornflower blue) and the glycan loop (red; residues 151 to 165) from another monomer (gray) are shown. **b** Amino acid sequence alignment of E proteins from different flaviviruses displaying sequence variation in the corresponding region of P31. Black arrows above the alignment represent secondary structure elements (β-strands) of ZIKV. The epitope region (P31) of anti-ZIKV mAbs is boxed. The red triangle denotes N-linked glycosylation sites. The positions where the sequences of tested viruses possessed residues identical to those of ZIKV/H/PF/2013 strain are indicated with dots.

According to the high-resolution structures of mature ZIKV (PDB: 5IRE)^18,19^, the “P31” epitope is positioned on the “150 glycan loop” that contains the N154 glycosylation site and is located between β-strands E_0_ and F_0_ of EDI. This epitope is adjacent to E protein dimer interface and fusion loop of the neighboring E protein (Fig. 4a), and appears to be highly exposed on the mature virion surface.

It is interesting that the mAbs targeting the glycan loop bind the synthetic P31 peptide which is non-glycosylated. To determine whether glycosylation of the epitope affects mAb binding, we compared *E.coli*-produced non-glycosylated E80 protein with insect S2 cell-expressed glycosylated E80 in ELISA with mAb 5F8 as the detection antibody. As shown in Supplementary Fig. S3a, both forms of E80 exhibited comparable 5F8-binding activities. We also examined whether mAb 5F8 directly binds ZIKV virions in ELISA. It was found that mAb 5F8 strongly reacted with purified inactivated ZIKV but not the control antigen prepared from uninfected Vero cells (Supplementary Fig. S3b). Together, these data indicate that glycosylation state does not significantly affect the “P31” epitope recognition by mAb 5F8.

### Comparison of the P31 epitope sequences among different flaviviruses

Amino acid sequence alignment of E proteins from different flaviviruses showed that the mAb epitope (residues 151 to 165) is identical among all Asian-lineage ZIKV strains (H/PF/2013, Z1106033, SZ-WIV01, PRVABC-59, PAN-259364, FLR, and Brazil-ZKV2015) and is relatively conserved in African ZIKV strains (MR766, ARB7701, and ArD158084) with each of these African ZIKV strains having only a single residue variation at positions 156 or 158 within the epitope (Fig. 4b). However, significant sequence variations were found in the corresponding regions of other flaviviruses including DENV1, DENV2, DENV3, DENV4, Japanese encephalitis virus (JEV), West Nile virus (WNV), and yellow fever virus (YFV) (Fig. 4b).

### Mechanism of mAb 5F8-mediated neutralization

**Fig. 5:**
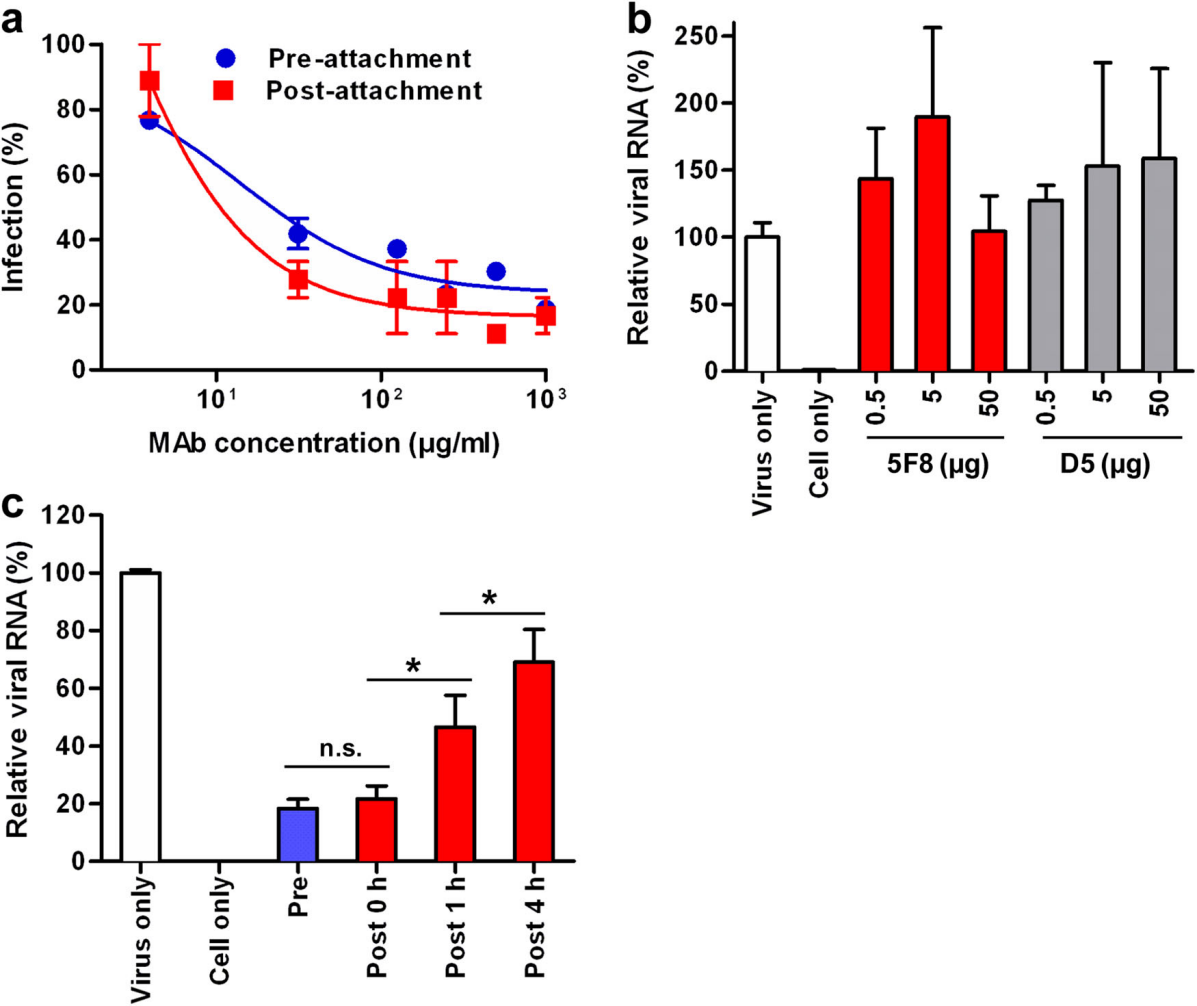
Mechanism of neutralization by mAb 5F8. **a** Pre- and post-attachment-inhibition assays. ZIKV was mixed with two-fold serial dilutions of mAb 5F8 before attaching to Vero cells (Pre-attachment) or were adsorbed to Vero cells followed by incubation with serially diluted 5F8 (Post-attachment). After washing, the plaque reduction assay was performed. Results are shown as the relative percentage of infection compared to the virus-only control with error bars illustrating SEM. **b** Attachment-inhibition assays. Different amounts of mAb 5F8 or D5 (negative control) were mixed with ZIKV at 4 °C for 1 h. The mixtures were then allowed to attach to Vero cells at 4 °C for 1 h. The cells were washed and collected, and viral RNA was then measured by real-time RT-PCR. **c** Influence of time of treatment with mAb 5F8 on neutralizing activity. Vero cells were incubated with the mAb 5F8-ZIKV mixture at 4 °C for 1 h (Pre) or ZIKV-bound Vero cells were allowed to incubate at 37 °C for the indicated times (0, 1, and 4 h) to facilitate virus entry prior to addition of mAb 5F8^49^. The cultures were then reincubated at 37 °C, and one day later the amount of viral RNA copies present in the cells was measured by real-time RT-PCR. Results are expressed as viral RNA levels in different treatment groups relative to that in the virus-only control group. Error bars represent SD. Statistical significance was indicated as follows: n.s., no significant difference (*P* ≥ 0.05); *P < 0.05; **P < 0.01.

As the five anti-ZIKV mAbs targeted the same epitope and possessed similar neutralization properties, we selected mAb 5F8, which had the highest neutralization potency (Table 1), as the representative mAb for subsequent mechanistic study. To determine the mode of action of mAb 5F8, we first performed pre- and post-attachment-inhibition assays. Serial dilutions of mAb 5F8 were incubated with ZIKV/SZ-WIV01 prior to or after virus attaching to Vero cells, and infection was determined using plaque reduction neutralization assay. We found that the neutralization efficiency of pre-attachment mAb treatment was not significantly different (*P* > 0.05) from that of post-attachment mAb treatment (Fig. 5a). This result shows that mAb 5F8 remained effective on the virions that had attached onto the cell surface.

Next, we performed attachment-inhibition assay to determine whether mAb 5F8 pretreatment could block virus attachment onto susceptible cells. We found that, compared to the control (virus without mAb), 5F8 pretreatment did not decrease the amount of cell-bound virus (Fig. 5b). This result indicates that mAb 5F8 could not block viral attachment. Together, the above data suggest that mAb 5F8 mounts neutralization effects primarily at the post-attachment stage.

To further determine the time frame when mAb 5F8 was capable of exerting post-attachment inhibition, we performed time-of-addition experiments. ZIKV-bound Vero cells were incubated for different times at 37 °C to allow virus entry, followed by addition of mAb 5F8. For comparison, the mAb-ZIKV mixture was added to Vero cells for one hour binding at 4 °C. Intracellular viral RNA levels for all treatments were detected by real-time PCR 24 h after infection. As shown in Fig. 5c, addition of mAb 5F8 immediately after virus-bound cells were switched to 37 °C (*t* = 0 h) efficiently reduced viral RNA levels to 22%, relative to the virus-only control, and its inhibitory effect was equivalent to that of antibody pretreatment. The inhibitory efficiencies of 5F8 decreased significantly when the mAb was added to the cells at relatively late time points (1 or 4 h post-infection). These results suggest that mAb 5F8 may function through blocking a very early step of the post-attachment entry process.

### MAb 5F8 did not enhance DENV infection in vitro

**Fig. 6:**
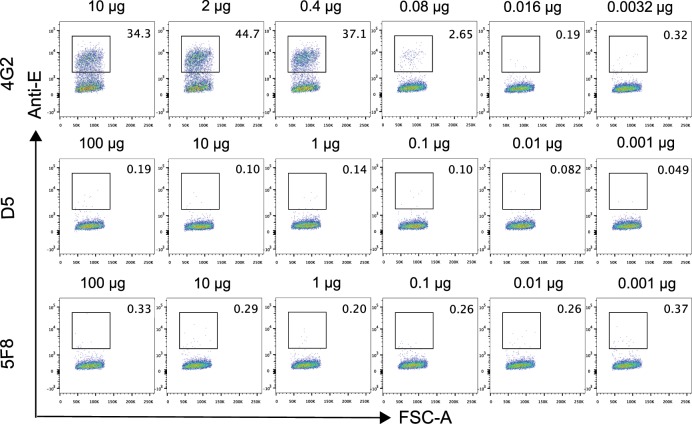
MAb 5F8 did not enhance DENV infection in vitro. DENV-2/NGC strain and serial dilutions of anti-ZIKV mAb 5F8, positive control mAb 4G2 or negative control mAb D5 were incubated prior to addition to FcγR-expressing K562 cells. Two days later, the cells were harvested, fixed, stained for viral antigen with Alexa Fluor 488-conjugated mAb 4G2 and then analyzed by flow cytometry. Antibody (4G2, D5, 5F8) amounts used are shown above each image. Results shown are representative of two independent experiments.

ADE of heterologous infections is a frequently observed phenomenon for anti-flavivirus antibodies^27,39^. In fact, it has been shown that some of the anti-ZIKV antibodies could enhance DENV infection in cell cultures and in mouse models^23,40^. We examined whether mAb 5F8 could also promote DENV infection by performing in vitro ADE assays. As shown in Fig. 6, the known cross-reactive ADE-inducing mAb, 4G2, strongly increased DENV2 infection at the antibody doses ranging from 10 to 0.4 µg; in contrast, no significant enhancement of DENV2 infection was observed for mAb 5F8 and the irrelevant control mAb D5, regardless of the antibody dose used (between 100 and 0.001 µg). These data demonstrate that mAb 5F8 does not enhance DENV2 infection in vitro.

### MAb 5F8 protects mice against lethal ZIKV challenge

**Fig. 7:**
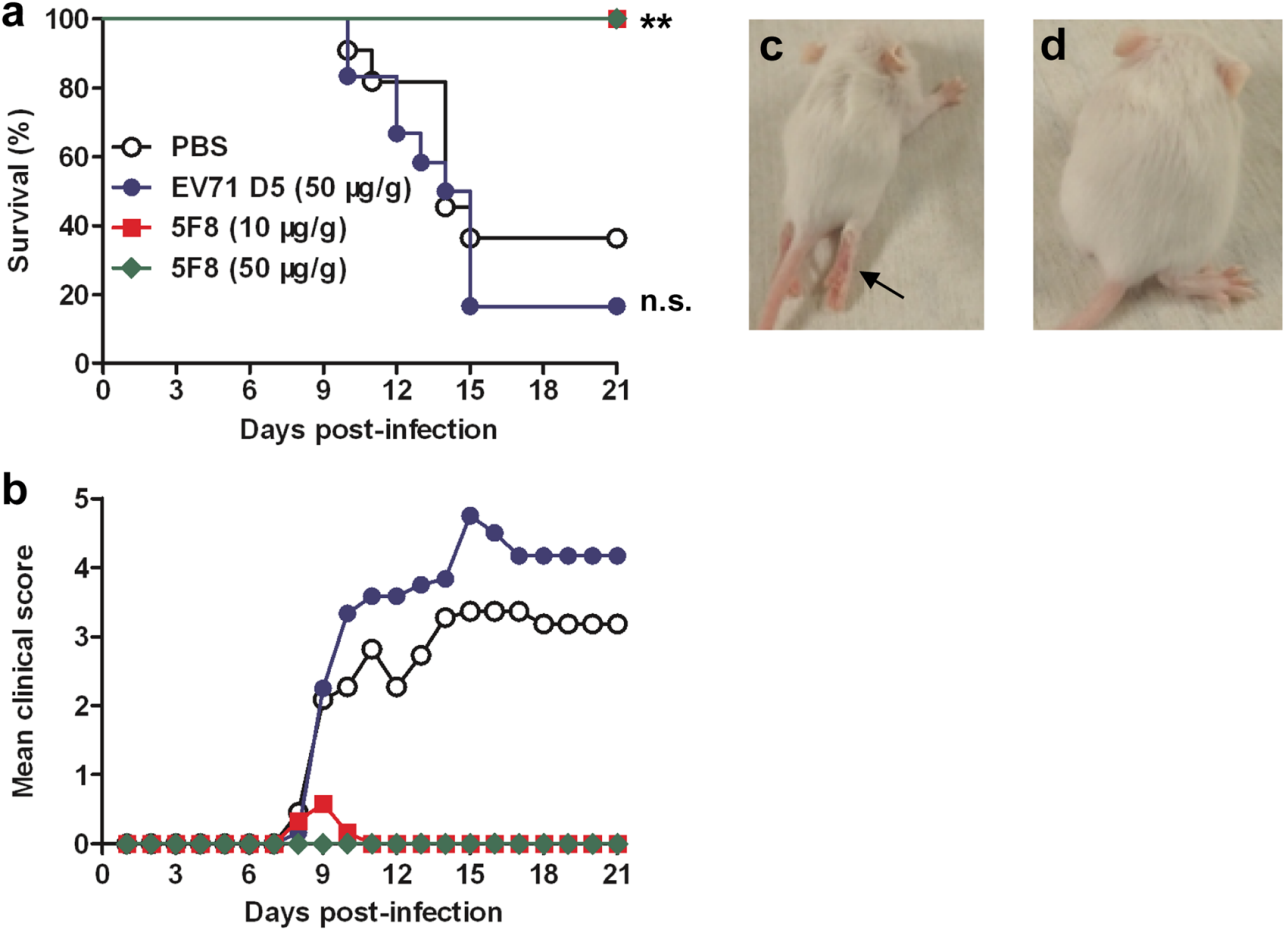
Protective efficacy of mAb 5F8 in a neonatal mouse model of ZIKV infection. Groups of one-day-old suckling ICR mice (n = 11–12/group) were inoculated i.p. with PBS, 10 µg/g of 5F8, 50 µg/g of 5F8, or 50 µg/g of control mAb D5, respectively, followed 24 hours later by i.p. injection with the mouse-adapted ZIKV strain ZIKV/MAV-HQ. (**a**) Survival and (**b**) clinical score were then monitored daily for 21 days following challenge. Clinical scores were graded as follows: 0, healthy; 1, reduced mobility; 2, limb weakness or toe walking; 3, tremors or imbalance; 4, paralysis; 5, moribund or death. **c**, **d** Representative mice treated with control mAb D5 (**c**) or anti-ZIKV mAb 5F8 (**d**) at 12 days post-infection. The black arrow indicates limb paralysis. Statistical significance was indicated as follows: n.s., no significant difference (*P* ≥ 0.05); **P < 0.01.

The protective efficacy of mAb 5F8 was assessed in an established mouse model^34^. Groups of one-day-old ICR mice were administrated i.p. with mAb 5F8 (10 or 50 µg/g body weight), or isotype control antibody D5 (50 µg/g body weight), or PBS and one day later infected with a mouse-adapted ZIKV strain, ZIKV/MAV-HQ^34^. All mice were subsequently monitored daily for clinical signs and survival for 21 days. As shown in Fig. 7, mice receiving PBS or mAb D5 gradually developed clinical signs such as reduced mobility, limb weakness, imbalance, and paralysis (indicated by the arrow in Fig. 7c), and the final survival rates of the PBS and D5 groups were 36.36% and 16.67%, respectively. In contrast, treatment with a single dose of mAb 5F8 resulted in 100% protection from death. All 5F8-treated mice were free of apparent clinical signs except one mouse in the low-dose (10 µg/g) group developed transient paralysis (Fig. 7). These results demonstrate that mAb 5F8 could efficiently protect mice from lethal ZIKV challenge.

## Discussion

In the present study, we identified five ZIKV neutralizing mAbs, namely 3E8, 5F8, 5G3, 8A2, and 9C3. All of the five mAbs specifically recognized and neutralized Asian-lineage ZIKV strains but they were unable to cross-react with or cross-neutralize African-lineage ZIKV, nor closely related DENV2 (Fig. 1c and Table 1). This group of mAbs possessed strong neutralization potency with PRNT_50_ against ZIKV/SZ-WIV01 ranging from 5.7 to 26 µg/ml (Table 1). In addition, these mAbs did not bind to DENV2 (Fig. 1) and did not promote ADE of DENV2 infection (Fig. 6). Moreover, the representative mAb 5F8 was found to confer full protection in a mouse model of lethal ZIKV challenge (Fig. 7). Collectively, these data show that these mAbs are strong candidates for further development into mAb-based therapeutic drugs for treating ZIKV infection. In addition, the unique binding property of these mAbs renders them applicable for developing rapid diagnostic kits that can distinguish Asian-lineage ZIKV from African-lineage ZIKV as well as from other flaviviruses such as DENV.

The epitope recognized by this group of neutralizing monoclonal antibodies described in this paper has not been reported previously. Epitope mapping revealed that all five ZIKV neutralizing mAbs bound the same linear epitope (P31 epitope: MIVNDTGHETDENRA) located on the glycan loop of EDI (Figs. 3 and 4). Sequence alignment showed that the epitope sequences were completely conserved among all Asian-lineage ZIKV strains analyzed here (Fig. 4b), suggesting that the five mAbs are potentially capable of neutralizing all contemporary ZIKV Asian strains. Thus, the five mAbs form a unique mAb group that targets the P31 epitope and possesses broadly neutralizing activity on Asian-lineage ZIKV. It is also found that the P31 epitope sequence in African-lineage ZIKV strains differed from that of the Asian-lineage strains by only one amino acid. Compared to Asian-lineage strains, African-lineage strain MR766, which the mAbs were unable to neutralize and recognize (Fig. 2b, c), had a single amino acid variation at position 158 (H → Y) (Fig. 4b). Therefore, H158 appeared to be an important residue in the P31 epitope for mAb binding. We should point out that our epitope mapping work was based on mAb binding to synthetic peptides which only display linear epitopes. The exact binding mode and detailed interaction of our mAbs with the epitope on ZIKV virions remain to be established perhaps by high-resolution structural analysis of virion-mAb complexes.

The P31 epitope resides on the glycan loop of EDI. As this epitope is outside of the EDIII domain which harbors receptor binding sites and mediates virus attachment to susceptible cells^41,42^, it is likely that P31-targeting mAbs would not be able to block virus attachment. According to the ZIKV structure models^18,43^, the EDI glycan loop is adjacent to fusion loop of neighboring E proteins within E dimers on mature virions. Therefore, antibody binding to the glycan loop might create steric hindrance for the access to the fusion loop, leading to inhibition of virus entry and infection. Consistent with this speculation, we found that the representative mAb, 5F8, did not block ZIKV attachment to the cell surface, it rather exerted inhibitory function at an early phase of the post-attachment viral entry process (Fig. 5). However, it remains to be determined whether mAb 5F8 indeed blocks the membrane fusion step during viral entry.

It is worth noting that all five neutralizing mAbs generated in this study were against the EDI glycan loop and no EDIII-targeting neutralizing mAb was recovered. This finding is surprising, as other groups have reported that neutralizing mAbs isolated from ZIKV-infected mice and humans were directed predominant at EDIII or fusion loop^30,31^. We speculate that the immunogen we used to generate mAbs, recombinant E80 protein, may significantly differ from its counterpart on virions in conformation and epitope exposure, leading to distinct mAb production profiles. However, this speculation remains to be verified by experimentation. Nonetheless, our results showed that EDI domain indeed contains epitopes for protective antibody production, which therefore should be taken into consideration when designing recombinant protein- or epitope-based ZIKV vaccines.

In summary, the present study generates a group of mAbs that exhibited broad neutralization in vitro and effective protection in vivo against Asian-lineage ZIKV infections. This study also defines a unique linear neutralization epitope located on the highly conserved EDI glycan loop. Our work thus provides valuable reagents and important information for developing ZIKV diagnostics, vaccines and mAb therapies.

## Materials and methods

### Cells and viruses

Vero cells and mouse myeloma cell line SP2/0 were cultured as described previously^35^. ZIKV strains used in this study include SZ-WIV01 (GenBank ID: KU963796)^44^, PRVABC59 (GenBank ID: KX377337), COL/FLR/2015 (FLR; GenBank ID: KX087102), MR766 (GenBank ID: KU720415), and a mouse-adapted ZIKV strain MAV-HQ^34^. DENV2 strain New Guinea C (DENV2/NGC) (GenBank ID: AF038403) has been described previously^45^. All viruses were propagated in Vero cells. Virus titers were measured by plaque assay and expressed as plaque forming units (PFUs) per mL as described previously^34^.

### Antigens and polyclonal antibodies

ZIKV (Z1106033 strain; GenBank ID: KU312312) E80 and EDIII proteins (residues 1 to 409, and 297 to 406 of E protein, respectively) were separately produced in *Drosophila* S2 cells as described previously^34^. DENV E80 protein (residues 1 to 400 of E protein) derived from DENV2 strain 16681 (GenBank ID: KU725663) was generated in S2 cells using identical protocols to those described above for ZIKV protein expression. Purified E80 and EDIII proteins were quantified by Bradford assay. Polyclonal antibodies against ZIKV E80 were prepared in our laboratory from BALB/c mice immunized with purified ZIKV E80 protein.

### Preparation of anti-ZIKV mAbs

The animal studies were approved by the Institutional Animal Care and Use Committee at the Institut Pasteur of Shanghai. Mice were obtained from Shanghai Laboratory Animal Center (SLAC, China).

Prior to immunization, purified ZIKV E80 protein (10 μg/dose) was formulated with aluminum hydroxide adjuvant (500 μg/dose; Invivogen, USA). Six-week-old female BALB/c mice were intraperitoneally (i.p.) immunized four times at 2-week intervals with the aluminum-adsorbed E80 antigen. Serum samples were collected from each mouse two weeks after the last vaccination and subjected to neutralization assay as described below to determine neutralizing antibody titers against ZIKV. The mouse with the highest neutralization titer was boosted intravenously with 50 μg of ZIKV E80 protein. Three days later, spleen cells from the selected mouse were harvested and fused with SP2/0 myeloma cells in the presence of polyethylene glycol (PEG) 1450 (Sigma, USA). The resultant fused cells were cultured for nine days in HAT (hypoxanthine, aminopterin and thymidine; Sigma) selection medium. Next, hybridoma supernatants were screened by ELISA as described below for their reactivity with ZIKV E80 protein. After screening 2–3 times, final hybridoma cell lines were obtained. MAbs were purified using protein G affinity column (Hitrap™, GE Healthcare, USA) as described previously^35^.

### Neutralization assay

Neutralizing activities of E80-immunized mouse sera, hybridoma culture supernatants, and purified mAbs against ZIKV were measured by plaque reduction neutralization test (PRNT) as described previously^34^. Briefly, 100 µL of two-fold serially diluted tested samples (sera, culture supernatant, or mAbs) were mixed with 100 PFU of ZIKV and incubated at 37 °C for 1 h. The mixtures were added to confluent Vero cells grown in 24-well plates and incubated at 37 °C for 1 h. Then supernatants were removed, and cell monolayers were overlaid with agarose overlay medium. After ~72 h of incubation at 37 °C, cells were fixed and stained with crystal violet and plaques were then counted. Neutralizing activities of purified mAbs against DENV were determined using similar procedures as described above. 50% plaque reduction neutralization titers (PRNT50) were calculated by nonlinear regression analysis using the GraphPad Prism 5.0 software.

### ELISA for screening of hybridomas and characterization of mAbs

To screen hybridomas, micro-ELISA plates (Nunc, USA) were coated overnight at 4 °C with 200 ng/well of ZIKV E80 protein, and blocked with 5% milk in PBS-Tween20 (PBST). 50 μL of undiluted hybridoma culture supernatants was added to the plates and incubated at 37 °C for 2 h. Plates were then washed with PBST and incubated with horseradish peroxidase (HRP)-conjugated anti-mouse IgG (Sigma, USA). After washes and color development, absorbance at 450 nm was measured.

Immunoglobulin isotypes of the mAbs were measured using SBA Clonotyping^TM^ System/HRP ELISA kit (Southern Biotech, USA) according to manufacturer’s instructions.

To measure binding properties of these mAbs, microplates (Nunc) were coated at 4 °C overnight with 200 ng/well of ZIKV E80, or DENV2 E80, and then blocked with 5% milk in PBST. Next, 50 μL/well of serially diluted anti-ZIKV mAbs, anti-EV71 mAb D5 (isotype control)^35^ or anti-DENV mAb D1-11 (Santa Cruz Biotechnology, USA) were added and incubated at 37 °C for 2 h. After washing with PBST, plates were incubated with HRP-conjugated anti-mouse IgG (Sigma). After color development, absorbance at 450 nm was measured.

### BLI assay

Binding affinities of anti-ZIKV mAbs towards ZIKV E80 were determined by BLI using Octet^®^ RED96 System (Pall FortéBio, USA) according to a previously described protocol^46^. Briefly, ZIKV E80 protein was biotinylated using EZ-Link™ Sulfo-NHS-LC-LC-Biotin (Thermo Fisher Scientific) and bound to streptavidin biosensors (Pall FortéBio) for 15 min. Then E80-immobilized biosensors were exposed to five-fold serially diluted mAb samples for 15 min to yield association curve and then allowed to dissociate for 15 min. Equilibrium dissociation constants (KD) for the E80/mAb interaction were calculated using Octet data analysis software (Pall FortéBio).

### Flow cytometry assay

Confluent Vero cells in six-well plates were infected with ZIKV/SZ-WIV01 or ZIKV/MR766 at a multiplicity of infection of 0.1, or with DENV2/NGC at a MOI of 0.5. Two days after infection, cells were detached from culture plates with trypsin-EDTA (Gibco™, Thermo Fisher Scientific, USA), fixed with 4% paraformaldehyde, and permeabilized with permeabilization buffer (eBioscience, USA). Then cells were stained with 1 μg/ml anti-ZIKV mAbs, anti-EV71 mAb D5 (isotype control)^35^ or flavivirus cross-reactive mAb 4G2 (Novus Biologicals, USA)^36^. After washing with PBS, cells were incubated with Alexa Fluor 488-conjugated anti-mouse-IgG (Proteintech, USA) and then analyzed by flow cytometry using a LSR II flow cytometer (BD Biosciences, USA).

### Western blotting

Western blotting was carried out as described previously^35^ but with minor modifications: E80 and EDIII proteins of ZIKV were separated on 15% SDS-PAGE gels and transferred onto PVDF membranes, and anti-ZIKV mAbs or anti-ZIKV-E80 sera served as the detection antibodies.

### Peptide ELISA

Epitopes for anti-ZIKV mAbs were mapped using peptide ELISA. Briefly, a total of 79 overlapping peptides covering the whole amino acid sequence of E80 protein of ZIKV/Z1106033 were synthesized by GL Biochem (Shanghai, China). Each peptide consists of 15 amino acid residues and overlaps with its adjacent peptides by 10 residues on both sides. An irrelevant HCV peptide HCV1^47^ was used as the negative control. Additionally, for fine epitope mapping, 15 variant peptides, each of which differs from the native sequence of peptide #31 by a single amino acid substitution, were synthesized. For peptide ELISA, microplates (Nunc) were coated with 2 µg/well of individual peptide in PBS at 4 °C overnight and then blocked with 5% milk in PBST. After washing with PBST, plates were incubated with 50 ng/well of anti-ZIKV mAbs at 37 °C for 2 h, followed by incubation with HRP-conjugated anti-mouse IgG (Sigma). After color development, absorbance at 450 nm was measured.

### Neutralization-inhibition assay

100 µL of serially diluted peptides (P31 and P1) were separately incubated with 100 µL (70 µg) of mAb 5F8 at 37 °C for 1 h. The peptide/mAb mixtures were then incubated with 100 µL (50 PFU) of ZIKV/SZ-WIV01 at 37 °C for 1 h. The mixtures (300 µL/well) were added to confluent Vero cells grown in 24-well plates and incubated at 37 °C for 1 h to allow infection. The supernatants were discarded, and 500 μL of fresh media was added. After 16 h culture, the supernatants were removed, and the amounts of viral RNA in cells were determined by real-time reverse transcription PCR (RT-PCR) using a previously described protocol^48^ with ZIKV-specific primers (ZIKV-ASF, 5′-GGTCAGCGTCCTCTCTAATAAACG-3′ and ZIKV-ASR, 5′-GCACCCTAGTGTCCACTTTTTCC-3′) and GAPDH-specific primers (forward primer, 5′-GTCTTCACCACCATGGAGAAGGC-3′; reverse primer, 5′-CATGGATGACCTTGGCCAGGGG-3′). Relative quantification of viral RNA was performed using the 2^−ΔΔCt^ method.

### Sequence alignment

Flaviviruses used for alignment include ZIKV/H/PF/2013 (GenBank ID: KJ776791), ZIKV/Z1106033 (KU312312), ZIKV/SZ-WIV01 (KU963796), ZIKV/PRVABC-59 (KX377337), ZIKV/PAN-259364 (KX156776), ZIKV/FLR (KX087102), ZIKV/Brazil-ZKV2015 (KU497555), ZIKV/MR766 (KU720415), ZIKV/ARB7701 (KF268950), ZIKV/ArD158084 (KF383119), DENV1/Hawaii (KM204119), DENV2/NGC (KM204118), DENV3/H87 (KU050695), DENV4/H241 (KR011349), JEV/KV1899 (AY316157), WNV/NY99 (DQ211652), YFV/ASIBI (AY640589). E protein sequences of these flaviviruses were aligned using BioEdit software.

### Pre- and post-attachment-inhibition assays

Capacities of the mAbs to inhibit ZIKV infection at pre- and post-attachment stages were assessed by two different assays. For pre-attachment assay, two-fold serial dilutions of anti-ZIKV mAb 5F8 were preincubated with 2000 PFU of ZIKV/SZ-WIV01 at 4 °C for 1 h. The mixtures were then added to prechilled Vero cells in 24-well plates and incubated at 4 °C for 1 h to allow attachment. After three washes with prechilled PBS, plaque reduction assay was performed as described previously^34^. For post-attachment assay, 2000 PFU of ZIKV/SZ-WIV01 was first incubated with cooled Vero cells at 4 °C for 1 h to permit viral attachment. Cells were rinsed to remove free virus and incubated with serially diluted 5F8 at 4 °C for 1 h. After washing, plaque reduction assay was performed as mentioned above.

To test the effect of time of mAb treatment on inhibitory activity, 100 PFU of ZIKV/SZ-WIV01 was mixed with 100 µg/mL mAb 5F8 and then added to prechilled Vero cells followed by incubation at 4 °C for 1 h (pretreatment); 100 PFU of ZIKV/SZ-WIV01 was allowed to bind to Vero cells at 4 °C for 1 h and then reincubated at 37 °C for different time points (0, 1, or 4 h) to facilitate virus entry before treatment with 100 ug/mL 5F8 (post-treatment). After further incubation at 37 °C for 24 h, the cells were washed once with PBS and collected, and viral RNA was then detected by real-time RT-PCR as described above. Relative quantification of viral RNA was performed using the 2^−ΔΔCt^ method.

### Attachment-inhibition assays

Different amounts (0.5 µg, 5 µg or 50 µg) of anti-ZIKV mAb 5F8 or anti-EV71 mAb D5 (negative control) were incubated with 5000 PFU of ZIKV/SZ-WIV01 at 4 °C for 1 h. The mixtures were then added to prechilled Vero cells and incubated at 4 °C for 1 h. After three washes with prechilled PBS, the cells were collected, and viral RNA was then determined by real-time RT-PCR as described above.

### ADE assay

2 × 10^5^ PFU of DENV-2/NGC was incubated with serial dilutions of anti-ZIKV mAb 5F8, anti-EV71 mAb D5 (isotype control) or flavivirus cross-reactive mAb 4G2 (positive control) at 37 °C for 1 h. The mixtures were then added to FcγR-expressing K562 cells plated at 2 × 10^5^ cells/well per 12-well plates. After incubation at 37 °C for 48 h, cells were harvested, fixed, and permeabilized, followed by staining for DENV antigen with 1 µg/ml Alexa Fluor 488-conjugated mAb 4G2. The percent of infected cells was determined by flow cytometry using a LSR II flow cytometer (BD Biosciences).

### In vivo protection assays

Groups of one-day-old ICR mice were i.p. administered with anti-ZIKV mAb 5F8 (10 or 50 µg/g body weight), anti-EV71 mAb D5 (isotype control; 50 µg/g body weight), or PBS. Twenty-four hours later, the suckling mice were inoculated i.p. with 1 PFU of the mouse-adapted strain ZIKV/MAV-HQ and then monitored daily for survival and clinical score for 21 days. Clinical scores were graded as follows: 0, healthy; 1, reduced mobility; 2, limb weakness or toe walking; 3, tremors or imbalance; 4, paralysis; 5, moribund or death.

### Statistical analysis

All statistical analyses were performed using GraphPad Prism version 5. Statistical comparisons of data between groups were analyzed by Student’s *t* test.

## Supplementary information

Supplementary figures

## Supplementary information

**Supplementary Information** accompanies the paper at (10.1038/s41421-019-0140-8).
