# Supplementary material for: A new class of broadly neutralizing antibodies that target the glycan loop of Zika virus envelope protein
Source: Cell Discov. 2020 Feb 4;6:5. doi: 10.1038/s41421-019-0140-8 (PMC6997156; doi:10.1038/s41421-019-0140-8)
Supplement: Supplementary file 1 — Supplementary figures [file 41421_2019_140_MOESM1_ESM.pdf]

Supplementary Information

Clone	OD450nm
1C11	1.10
1D3	0.61
1E2	0.67
3D1	0.69
3E8	0.64
3H4	0.97
4A12	0.60
4B10	0.65
4C5	1.08
4C10	1.00
4F5	0.86
4G4	0.59
5B3	0.51
5F8	1.08
5G3	0.72

Clone	OD450nm
6A1	0.57
6C1	0.98
6E9	0.44
6F9	0.98
6G7	1.16
6H4	1.06
6H5	0.80
8A2	0.49
8G8	0.98
9A4	0.46
9B7	0.81
9C3	0.62
9E5	0.63
9E11	0.90
9G5	0.86

Supplementary Fig. S1. OD450 values of 30 ZIKV-specific hybridoma clones.

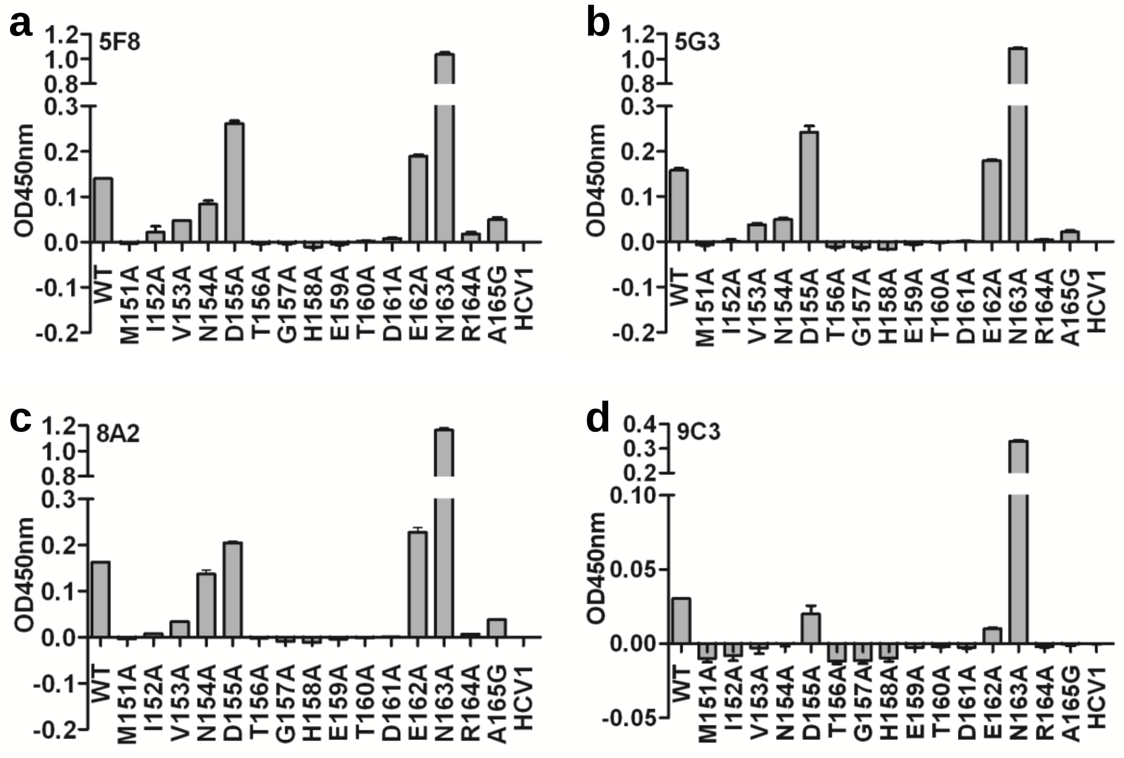

Supplementary Fig. S2. Fine epitope mapping of mAbs by scanning mutagenesis. Fifteen peptides with a single amino acid substitution at each position of P31 were tested for their reactivities with 5F8 **(a)**, 5G3 **(b)**, 8A2 **(c)**, 9C3 **(d)** by peptide ELISA. WT, wild type P31. Peptide variant nomenclature: first letter = original amino acid; number = position in ZIKV E protein; second letter = mutant amino acid. Error bars represent SEM.

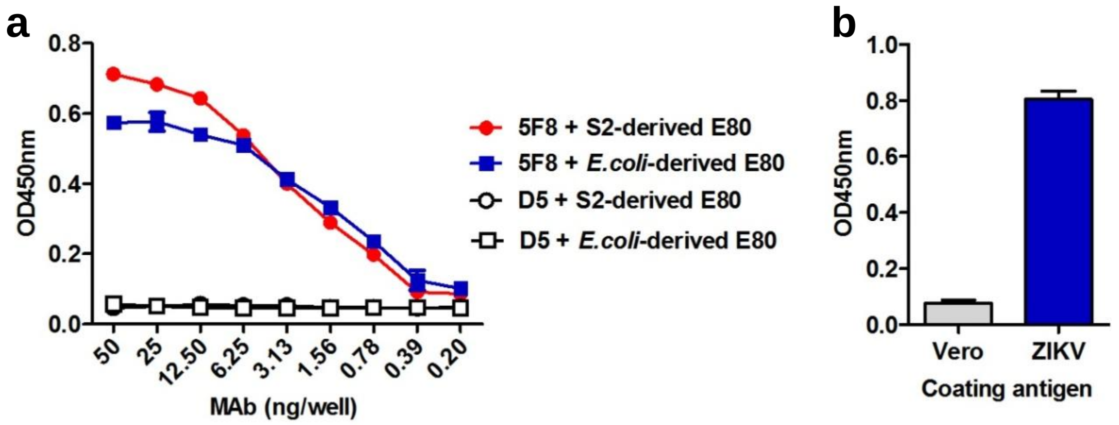

Supplementary Fig. S3. Binding of mAb 5F8 to different forms of ZIKV E protein. **(a)** Reactivity of mAb 5F8 with glycosylated and non-glycosylated recombinant E80 proteins in ELISA. Wells of the ELISA plates were coated with 200 ng/well of E. coli-expressed (non-glycosylated) E80 or S2 cell-expressed (glycosylated) E80. Serially diluted mAb 5F8 or isotype control antibody D5 were used as the primary antibody. Data are OD450nm values (mean ± SD) of triplicate wells. **(b)** Binding of mAb 5F8 with ZIKV virions. Wells of the ELISA plates were coated with 20 ng/well of purified inactivated ZIKV or the control antigen prepared from uninfected Vero cells. MAb 5F8 was used as the detection antibody in the ELISA. Data are OD450nm values (mean ± SD) of triplicate wells.
